# Awareness of Dental Professionals of the Relationship Between Prosthetic Pressure Lesions and Infective Endocarditis: A Cross-Sectional Observational Study

**DOI:** 10.3390/clinpract16070129

**Published:** 2026-07-10

**Authors:** Francesco Saverio Ludovichetti, Serena Zanatta, Davide Scettri, Valentina Brun, Sergio Mazzoleni, Edoardo Stellini, Christian Bacci

**Affiliations:** Dentistry Section, Department of Neurosciences, University of Padova, 35128 Padova, Italy

**Keywords:** prosthetic pressure lesions, infective endocarditis, removable dentures, transient bacteremia

## Abstract

**Background**: Infective endocarditis is a serious disease affecting cardiac tissues and may arise following episodes of transient bacteremia. Oral prosthetic pressure lesions, characterized by mucosal ulcerations, may facilitate the entry of pathogenic microorganisms into the systemic circulation. In predisposed individuals, the presence of such lesions may therefore contribute to the development of infective endocarditis. The objective of this study was to assess dental professionals’ knowledge, awareness, and self-reported clinical practices related to the possible relationship between oral prosthetic pressure lesions and infective endocarditis. **Materials and Methods**: A cross-sectional observational study was conducted using an anonymous online questionnaire specifically developed for this study and subjected to preliminary content validation and pilot assessment before administration. The survey was distributed online to oral health professionals working in the Italian public and private sectors; for the present analysis, only complete responses provided by licensed dental professionals practicing in Italy were included. **Results**: Among the 268 dental professionals analyzed, an overall limited awareness of the possible relationship between oral prosthetic pressure lesions and infective endocarditis emerged. Only 16.8% of participants reported being familiar with the most recent guidelines for the prevention of infective endocarditis. **Conclusions**: The findings suggest the need to strengthen continuing education, improve familiarity with current infective endocarditis prevention guidelines, and promote more structured clinical protocols for the assessment and management of prosthetic pressure lesions in patients at risk.

## 1. Introduction

Infective endocarditis (IE) is a serious condition involving the endocardial structures and is associated with substantial morbidity and mortality. Its pathogenesis is closely linked to episodes of transient bacteremia, through which microorganisms enter the bloodstream and may colonize damaged endocardial surfaces in predisposed individuals [1,2]. Although it is a relatively rare condition, infective endocarditis represents a significant clinical problem due to the high complexity of its management and the possible associated complications. The most recent epidemiological data indicate an estimated annual global incidence of around 13.8 cases per 100,000 inhabitants, with an increasing trend in relation to the aging of the population and the growing complexity of patients [3]. Transient bacteremia is a central element in the pathogenesis of infective endocarditis; it is defined as the episodic passage of microorganisms into the bloodstream starting from a peripheral focus. This phenomenon allows pathogens to reach endocardial structures and adhere to previously damaged surfaces, favoring the establishment of the infectious process [2]. Traditionally, the onset of bacteremic episodes has been associated with invasive procedures, including dental ones; however, more recent evidence indicates that bacteremia can occur even in the absence of clinical maneuvers, in relation to local conditions that compromise the integrity of mucosal barriers [2,4,5]. In this context, the concept of repeated exposure to low-level bacteremic episodes takes on relevance, which could contribute to the development of infective endocarditis in predisposed subjects [2,3,6,7].

The oral cavity contains a complex microbiota that may contribute to systemic consequences when local barriers are disrupted. In particular, denture-related prosthetic pressure lesions may result in persistent or recurrent mucosal ulceration, thereby compromising the integrity of the oral mucosal barrier. From a biological perspective, this condition may facilitate the passage of oral microorganisms into the bloodstream and contribute to episodes of transient bacteremia, especially in the presence of local inflammation or biofilm accumulation. Since transient bacteremia is a recognized element in the pathogenesis of infective endocarditis, these lesions may be clinically relevant in predisposed individuals. Robust evidence supports the broader link between oral bacteria and systemic infectious conditions, including through bacteremia and inflammatory pathways [1,8,9]. Although direct evidence specifically linking prosthetic pressure lesions to infective endocarditis remains limited—which constitutes the rationale for this study—the recent literature reinforces the biologic plausibility of this association. Therefore, improved attention to these lesions is relevant in the preventive management of patients at increased risk of infective endocarditis [8,9]. Removable dental prostheses represent a widely used rehabilitation solution, but their use may be associated with the onset of local alterations to the oral mucosa, especially in the presence of prosthetic inconsistencies, instability of the product or prolonged use over time. Among the most frequent complications are oral prosthetic pressure lesions, characterized by ulcerations of the mucosa of varying severity, often localized in areas subjected to repeated mechanical loads [10,11,12,13,14]. The presence of ulcers causes a compromise of the integrity of the mucosal barrier, favoring the translocation of microorganisms resident in the oral cavity towards the systemic circulation and increasing the risk of transient bacteremia episodes [8,15]. Unlike acute traumatic events, prosthetic pressure lesions can persist over time or manifest themselves in a recurrent form, configuring a condition of repeated bacteremic exposure, potentially relevant in predisposed subjects [7,11,16]. Despite the biological plausibility of this mechanism, the possible contribution of oral prosthetic pressure lesions to the development of infective endocarditis is poorly investigated in the literature, particularly with regard to preventive and interceptive aspects in daily dental practice [16,17]. Oral prosthetic pressure lesions, although relatively frequent events in removable prosthesis wearers, seem to receive little attention in terms of possible systemic implications and preventive management, as emerges from the available literature [10,11,16]. Given the often persistent or recurrent nature of these lesions, their early interception and correct clinical assessment take on a significant importance in the context of prevention [11,15,18]. In this scenario, there is a clear lack of structured data that systematically evaluates the level of awareness of dental professionals regarding the possible relationship between oral prosthetic pressure ulcers and infective endocarditis, as well as the ways in which this awareness is reflected in daily clinical practice [2,16]. This knowledge gap constitutes the rationale for the present observational study.

The primary objective of this study was to assess the knowledge, awareness, and self-reported clinical practices of dental professionals regarding the possible relationship between oral prosthetic pressure lesions and infective endocarditis. Specifically, the study explored theoretical knowledge, approaches to the recognition and management of prosthetic pressure lesions, and attention to medical history taking and systemic risk assessment in routine clinical practice.

## 2. Materials and Methods

### 2.1. Study Design

A cross-sectional observational study was conducted using an anonymous online questionnaire to assess dental professionals’ awareness of the potential relationship between oral prosthetic pressure ulcers and infective endocarditis.

#### 2.1.1. Sample and Eligibility Criteria

The questionnaire was administered to dental professionals working in the Italian professional context, both in the public and private sectors. Participation in the study was voluntary and anonymous, without any financial incentive. A total of 356 questionnaires were collected. For the purposes of statistical analysis, only responses from dental professionals were considered, for a total of 268 questionnaires. Therefore, questionnaires completed by dental students or healthcare professionals not related to dentistry, as well as any incomplete or duplicate questionnaires, were excluded from the analysis.

#### 2.1.2. Survey Instrument

The questionnaire, developed specifically for the study, consisted of 18 questions (Table 1), structured as follows:

- Seven dichotomous questions;
- Seven multiple-choice questions, with the option to select more than one answer;
- Four Likert-scale questions.
- The questions were designed to investigate three main areas:
- demographic and professional data, including age, work environment, years of professional experience, methods of medical history update and ongoing training;
- clinical practices related to the recognition, treatment and prevention of oral prosthetic pressure lesions;
- theoretical and scientific knowledge regarding oral prosthetic pressure lesions, transient bacteremia, infective endocarditis and general indications for antibiotic prophylaxis. The questionnaire was specifically developed for this study based on the study objectives and on the relevant literature concerning infective endocarditis, transient bacteremia, denture-related pressure lesions, and related preventive clinical practices. Before dissemination, the questionnaire was reviewed by experts in the dental and academic fields to assess the relevance, clarity, and comprehensiveness of the items. A preliminary pilot assessment was also performed to verify the comprehensibility and consistency of the questions, and minor wording modifications were introduced accordingly. Therefore, the questionnaire underwent a preliminary content validation process before its final administration.

The questionnaire was created using the Google Forms platform and distributed online through digital channels dedicated to the profession (social media and email). Table 1 reports the questionnaire items and response options, whereas the main response distributions are presented in Table 2 and described in the Results section, Section 3.5.

### 2.2. Data Collection and Analysis

The collected data were analyzed using descriptive statistics, expressed in terms of absolute frequencies and percentages.

The threshold for statistical significance was set at *p <* 0.05. Statistical analyses and data graphical representation were performed using R software (R foundation for statistical computing, version 4.4.1, Vienna, Austria).

### 2.3. Ethical Considerations

The study was observational and non-interventional in nature. Participation was voluntary and completely anonymous; all participants were informed of the study’s objectives and how their data would be processed, in compliance with the European General Data Protection Regulation (GDPR 679/2016). Given the nature of the study, approval by an Ethics Committee was not required.

## 3. Results

### 3.1. Sample Characteristics

A total of 268 questionnaires completed by dental professionals working in the Italian professional context were analyzed, selected from an initial total of 356 responses collected. Among the 268 included participants, 95.9% worked in private practice and 4.1% in the National Health Service. The detailed distribution of the main questionnaire responses is reported in Table 2.

Most of the sample practiced in the private sector (95.9%), while a minority (4.1%) worked within the National Health System (Italian “Sistema Sanitario Nazionale—SSN”). Years of professional experience were heterogeneously distributed, including both professionals with less than one year of experience and dental professionals with over ten years of practice.

A significant portion of the sample reported participating in continuing education activities, although the methods and levels of training varied.

### 3.2. Knowledge of Guidelines and Infective Endocarditis

Regarding knowledge of clinical recommendations, 45 out of 268 participants (16.8%) reported being aware of the most recent guidelines on the prevention of infective endocarditis. In addition, over 40% of the sample reported never having received specific training on infective endocarditis during their education or professional career. These findings descriptively indicate heterogeneity in the level of theoretical and scientific preparation regarding this topic.

### 3.3. Awareness of the Role of Transient Bacteremia

Responses regarding the role of transient bacteremia in the pathogenesis of infective endocarditis were heterogeneous. Overall, the distribution of responses showed variable levels of agreement and awareness among participants, indicating that this topic was not uniformly recognized across the sample.

### 3.4. Clinical Management of Oral Prosthetic Pressure Lesions

Most participants reported being able to clinically recognize oral prosthetic pressure lesions and to implement local management interventions, such as temporarily discontinuing denture use, relining the prosthesis, or adopting conservative measures. However, these descriptive findings should be interpreted as self-reported clinical attitudes and not as evidence of actual clinical behavior.

### 3.5. Assessment of Systemic Risk in Patients with Removable Dentures

Regarding systemic risk assessment, 53.4% of the participants who stated that they were aware of the correlation between infective endocarditis and dental procedures reported not considering the risk of infective endocarditis when performing treatments on patients with removable dentures. Similarly, medical history updating and systemic risk assessment in the presence of ulcerative lesions were not reported uniformly across the sample. These results provide a descriptive overview of self-reported attitudes within the study population.

## 4. Discussion

This observational study descriptively analyzed dental professionals’ awareness of the potential relationship between oral prosthetic pressure ulcers and infective endocarditis, highlighting relevant aspects of both theoretical knowledge and self-reported clinical practice. The results show that, despite widespread recognition of prosthetic-related injuries and their local management in daily practice, the systemic dimension of risk is frequently underestimated. This finding is consistent with existing evidence suggesting the role of the oral cavity as a potential source of transient bacteremia and, indirectly, systemic infections [1,8,17]. This established connection between oral bacteria and systemic infectious conditions underscores the clinical relevance of our findings.

A particularly notable finding concerns the limited knowledge of the current recommendations for the prevention of infective endocarditis, reported by only 16.8% of the sample. In the present study, reference to the most recent guidelines concerns the current international recommendations, particularly the 2023 European Society of Cardiology (ESC) Guidelines for the management of endocarditis. Earlier guideline documents were considered only as historical background and not as the main reference framework for the interpretation of participants’ knowledge. This finding suggests a significant training gap regarding a serious systemic disease, for which prevention plays a central role, especially in at-risk individuals, as clearly indicated by the main international guidelines [10,19]. The variability in the training of non-medical healthcare professionals in relation to infective endocarditis has already been described in the literature and represents a critical element in the translation of clinical recommendations into daily practice [9,17].

The most significant finding emerging from the study is the misalignment between self-reported knowledge and clinical behavior. Over half of the dental professionals who claim to be aware of the correlation between infective endocarditis and dental procedures report not considering the risk of endocarditis when treating patients with removable dentures.

Transient bacteremia, recognized as a central element in the pathogenesis of infective endocarditis [9,19], is in fact only partially understood and internalized in the clinical practice of the analyzed sample. While traditional attention is often focused on invasive dental procedures, oral prosthetic pressure lesions represent a potentially persistent or recurrent condition, capable of favoring repeated episodes of low-level bacteremia [1,11,16]. In this context, the underestimation of chronic lesions compared to acute events could contribute to a distorted perception of the real risk, especially in patients predisposed to developing infective endocarditis [17,20].

The heterogeneity of observed clinical practices and the lack of shared protocols suggest the need to strengthen specific training and promote a more structured approach to systemic risk assessment.

The present study has some limitations. The cross-sectional observational design does not allow causal relationships to be established, while the self-reported nature of the questionnaire may have introduced response bias. In addition, the sample was heavily skewed toward professionals working in private practice, with only a small proportion employed in the National Health Service. This imbalance may limit the representativeness of the sample and the generalizability of the findings to the broader population of dental professionals in Italy. Furthermore, the exclusively Italian professional context may limit the transferability of the results to other healthcare systems. Despite these limitations, the adequate sample size and the focus on a little-investigated topic give the study exploratory value and provide a basis for future observational investigations [17,21,22].

## 5. Conclusions

This descriptive study suggests that awareness among the included dental professionals regarding the potential relationship between oral prosthetic pressure lesions and infective endocarditis may be limited. Within the study sample, limited familiarity with current reference guidelines and a gap between local lesion management and consideration of systemic risk were observed.

Given the descriptive design and the non-probabilistic nature of the sample, these findings should be interpreted with caution and cannot be generalized to the broader population of dental professionals. Nevertheless, the results highlight the potential value of further studies and targeted educational initiatives aimed at improving awareness and preventive attention in patients at risk of infective endocarditis.

## Figures and Tables

**Table 1:** Questionnaire items and response options used in the survey.

Questions	Possible Answers
What is your profession?	• Dental hygienist\newline • Dental hygiene student\newline • Dentist
How many years have you been practicing this profession?	• Less than 1 year\newline • 1 to 3 years\newline • 3 to 10 years\newline • More than 10 years
Have you attended specific training courses in oral medicine and oral pathology?	• Yes\newline • No
If you answered “Yes” to the previous question, what type of training?	• Lectures during the degree program\newline • Training days/courses\newline • 1st-level Master’s program\newline • None
What type of facility do you work in?	• Private practice\newline • National Health Service (SSN)
How often is the patient’s medical history updated in your workplace?	• At every appointment\newline • Every 6 months\newline • Once a year\newline • When the patient reports changes
During your professional training/career, have you ever received specific training on infective endocarditis?	• Yes\newline • No
How would you rate your knowledge of infective endocarditis?	• Excellent\newline • Good\newline • Adequate\newline • Limited\newline • None
Are you aware of the association between infective endocarditis and dental procedures?	• Yes\newline • No
How decisive do you think the development of transient bacteremia can be in the onset of infective endocarditis?	• Very decisive\newline • Decisive\newline • Fairly decisive\newline • Not very decisive\newline • Not decisive at all
To the best of your knowledge, is antibiotic prophylaxis recommended for all patients at risk of infective endocarditis?	• Yes\newline • No
To the best of your knowledge, is antibiotic prophylaxis recommended for patients at risk of severe outcomes of infective endocarditis?	• Yes\newline • No
When you perform dental treatments on patients with removable dentures, do you take the risk of infective endocarditis into account?	• Yes\newline • No
Which factors do you think may increase the risk of endocarditis in a patient with removable dentures?	• Poor oral hygiene\newline • Invasive procedures\newline • Presence of cardiovascular diseases\newline • Denture-related pressure sore
How much do you agree with the following statement: “A denture-related pressure sore can cause transient bacteremia?”	• Strongly agree\newline • Agree\newline • Disagree\newline • Strongly disagree
How able do you feel to recognize lesions caused by denture-related pressure sores?	• Completely able\newline • Quite able\newline • Moderately able\newline • Slightly able\newline • Not able at all
In your opinion/knowledge, which factors predispose to the development of denture-related pressure sores?	• Ill-fitting dentures\newline • MRONJ/ONJ\newline • Radiotherapy and chemotherapy\newline • Prolonged denture wearing\newline • Xerostomia\newline • Poor home oral hygiene\newline • Diabetes\newline • Pollen allergies\newline • Inadequate diet
Based on your knowledge and experience, how can denture-related pressure lesions be managed?	• Relining the denture base/components\newline • Applying adhesive at the denture–mucosa interface\newline • Temporarily discontinuing denture use\newline • Prescribing a soft diet

**Table 2:** The main response distributions of the questionnaire were among the 268 included dental professionals.

Domain	Item	Response	*n* (%)
Work setting	Type of facility	National Health Service (SSN)	11 (4.1%)
Work setting	Type of facility	Private practice	257 (95.9%)
Knowledge of transient bacteremia	How decisive is transient bacteremia in the onset of infective endocarditis?	Not very decisive	13 (4.85%)
Knowledge of transient bacteremia	How decisive is transient bacteremia in the onset of infective endocarditis?	Fairly decisive	58 (21.64%)
Knowledge of transient bacteremia	How decisive is transient bacteremia in the onset of infective endocarditis?	Decisive	105 (39.18%)
Knowledge of transient bacteremia	How decisive is transient bacteremia in the onset of infective endocarditis?	Very decisive	92 (34.33%)
Antibiotic prophylaxis	Is antibiotic prophylaxis recommended for all patients at risk of infective endocarditis?	Yes	192 (71.73%)
Antibiotic prophylaxis	Is antibiotic prophylaxis recommended for all patients at risk of severe outcomes of infective endocarditis?	Yes	258 (96.27%)
Guideline-consistent knowledge	Coherent identification of the indication for antibiotic prophylaxis according to current recommendations	Yes	45 (16.8%)

## Data Availability

The raw data supporting the conclusions of this article will be made available by the authors on request.
